# Immune Response to *Mycobacterium tuberculosis* Infection in the Parietal Pleura of Patients with Tuberculous Pleurisy

**DOI:** 10.1371/journal.pone.0022637

**Published:** 2011-07-28

**Authors:** Gaetano Caramori, Lisa Lasagna, Angelo G. Casalini, Ian M. Adcock, Paolo Casolari, Marco Contoli, Federica Tafuro, Anna Padovani, Kian Fan Chung, Peter J. Barnes, Alberto Papi, Guido Rindi, Giuseppina Bertorelli

**Affiliations:** 1 Section of Respiratory Diseases, University of Ferrara, Ferrara, Italy; 2 Department of Clinical Medicine, Nephrology and Health Science, University of Parma, Parma, Italy; 3 Respiratory Diseases and Thoracic Endoscopy Unit, Parma Hospital, Parma, Italy; 4 Airways Disease Section, National Heart and Lung Institute, Imperial College London, London, United Kingdom; 5 Institute of Anatomic Pathology UCSC, Policlinico A. Gemelli, Roma, Italy; Postgraduate Institute of Medical Education and Research, India

## Abstract

The T lymphocyte-mediated immune response to *Mycobacterium tuberculosis* infection in the parietal pleura of patients with tuberculous pleurisy is unknown. The aim of this study was to investigate the immune response in the parietal pleura of tuberculous pleurisy compared with nonspecific pleuritis. We have measured the numbers of inflammatory cells particularly T-cell subsets (Th1/Th2/Th17/Treg cells) in biopsies of parietal pleura obtained from 14 subjects with proven tuberculous pleurisy compared with a control group of 12 subjects with nonspecific pleuritis. The number of CD3+, CD4+ and CCR4+ cells and the expression of RORC2 mRNA were significantly increased in the tuberculous pleurisy patients compared with the nonspecific pleuritis subjects. The number of toluidine blue+ cells, tryptase+ cells and GATA-3+ cells was significantly decreased in the parietal pleura of patients with tuberculous pleurisy compared with the control group of nonspecific pleuritis subjects. Logistic regression with receiver operator characteristic (ROC) analysis for the three single markers was performed and showed a better performance for GATA-3 with a sensitivity of 75%, a specificity of 100% and an AUC of 0.88. There was no significant difference between the two groups of subjects in the number of CD8, CD68, neutrophil elastase, interferon (IFN)-γ, STAT4, T-bet, CCR5, CXCR3, CRTH2, STAT6 and FOXP3 positive cells. Elevated CD3, CD4, CCR4 and Th17 cells and decreased mast cells and GATA-3+ cells in the parietal pleura distinguish patients with untreated tuberculous pleurisy from those with nonspecific pleuritis.

## Introduction

Tuberculosis is the second most important cause of death from infectious diseases in the world. From 1990–2003, the incidence of tuberculosis increased globally and currently more than one third of the world's population is infected with *Mycobacterium tuberculosis* [1].

Pleural Tuberculosis (PLTB) results from *M.tuberculosis* infection of the pleura and can be associated with pulmonary tuberculosis [2]. PLTB occurs in 4% of newly diagnosed cases of tuberculosis and its frequency differs among countries [2], [3]. The human immunodeficiency virus (HIV) pandemic has been associated with a doubling of the incidence of extrapulmonary tuberculosis, which has resulted in increased recognition of PLTB even in developed countries [4].

PLTB diagnosis depends on demonstration of *M.tuberculosis* in sputum, pleural fluid or pleural biopsy specimens [2], [4]. A thoracoscopic biopsy of parietal pleura is the most sensitive diagnostic test. Histological examination of pleural biopsy may demonstrate granulomatous inflammation, caseous necrosis and/or acid-fast bacilli [4]. Detection of *M.tuberculosis* DNA by polymerase chain reaction (PCR) establishes the PLTB diagnosis. In contrast, non specific pleuritis (NSP) is characterized by chronic inflammation and deposits of fibrin in the subpleural compartment [5].

The pathogenetic hypothesis of PLTB suggests that activated CD3+ and CD4+ T-helper type (Th) 1 cells, through the release of interferon gamma (IFN-γ) and other Th1 cytokines, activate macrophages to kill *M.tuberculosis*, whereas Th2 cytokines may antagonize this effect [4]. The tuberculous pleural fluid is rich in lymphocytes, particularly CD4+ T cells [6]. In addition many studies support the presence of a Th1 polarization in pleural fluid in PLTB [7]–[9] and the IFN-γ level measurement has been proposed as a method for PLTB diagnosis [4]. Activation of T regulatory cells (Tregs; identified by the expression of the transcription factor FOXP3) may avoid an excessive inflammatory response and/or may compromise the elimination of *M.tuberculosis*. Recent studies suggest an increased presence of Tregs in PLTB [10], [11]. Other studies, however, demonstrated that most clones show a Th0 cytokine profile (production of both IFN-γ and interleukin (IL)-4) in untreated patients. After 6 months of therapy and clinical healing, most clones show a Th1 profile [12]. Finally, some reports suggest the potential for a Th2 response [13], [14].

*M.tuberculosis* infection can also induce IL-17 producing T-cell subsets (Th17). The orphan nuclear receptor retinoic orphan receptor (ROR)γt and its human homologue RORC2 are selective markers for Th17 cells [15]. IL-17 is a potent inflammatory cytokine capable of inducing chemokine expression and cell recruitment into tissue. Both the IL-17 and the Th17 response to *M.tuberculosis* are largely dependent upon IL-23 [16]. Th1 and Th17 responses cross-regulate each other during infection and this may be important for the immunopathology of tuberculosis [16].

There are no studies investigating T-cell subpopulations in pleural biopsies obtained from PLTB patients and control groups. The aim of the present study was to investigate the inflammatory cell infiltrate (CD3, CD4 and CD8 T cells, macrophages, neutrophil and eosinophil granulocytes and mast cells) and a panel of Th1 (IFN-γ, STAT4, T-bet, CCR5 and CXCR3+ cells), Th2 (CCR4, CRTH2, GATA-3 and STAT6+ cells), Tregs (FOXP3+ cells) and Th17 (RORC2 mRNA) markers in parietal pleural biopsies from PLTB patients compared with a NSP control group.

## Results

### Histochemistry count for mast cells and eosinophil granulocytes

**Figure 1:**
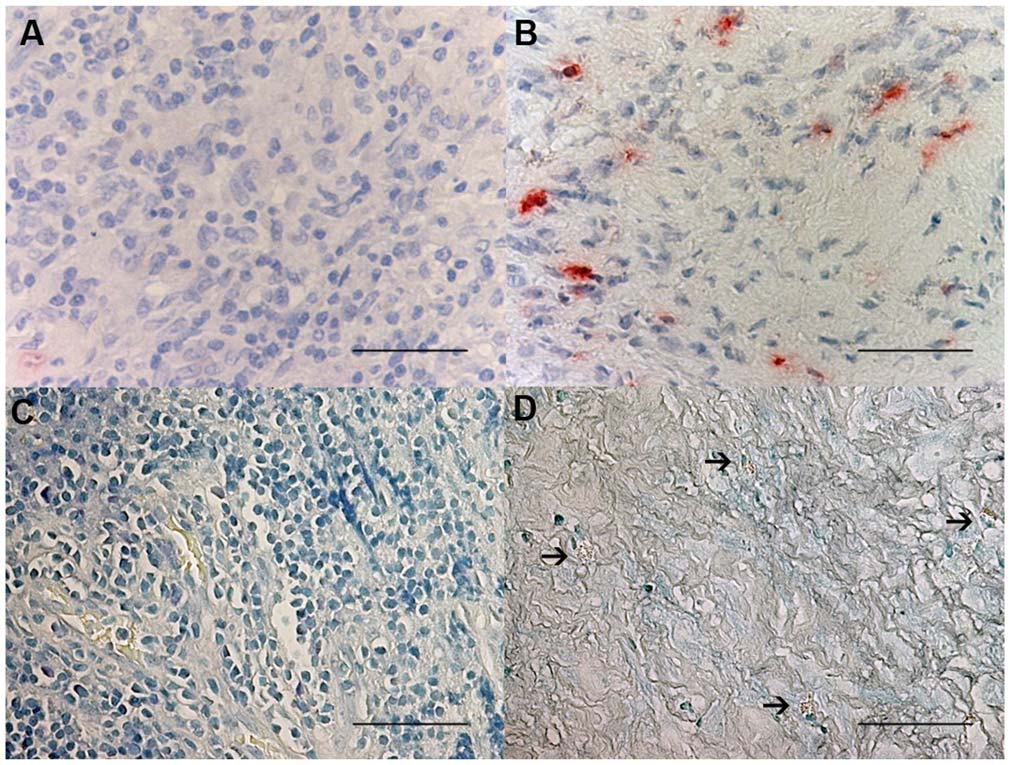
Photomicrographs showing the parietal pleura stained for mast cells using immunostaining with an anti-tryptase antibody (A and B) or toluidine blue histochemical staining (C and D). Tryptase+ cells are stained in red and toluidine blue+ cells are stained in blue. Results are representative of those from 14 patients with PLTB (A and C) and 12 patients with NSP (B and D). Original magnification: 400×. The scale bar represents 50 µm.

**Figure 2:**
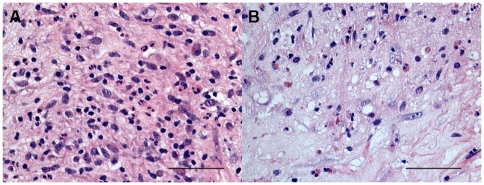
Photomicrographs showing the parietal pleura stained for eosinophils using hematoxylin and eosin (H/E) histochemical staining (A and B). Eosinophil granulocytes+ cells are stained in pink. Results are representative of those from 14 patients with PLTB (A) and 12 patients with NSP (B). Original magnification: 400×. The scale bar represents 50 µm.

The number of toluidine blue+ cells was significantly decreased in PLTB patients compared with the NSP subjects (1.26±0.91 vs 51.96±29.14, p<0.009, Table 1 and Figure 1), whereas the number of eosinophil granulocytes was not significantly different between the two groups (100.0±27.7 vs 65.2±19.3 for PLTB and NSP respectively, Table 1 and Figure 2).

**Table 1:** Quantification of inflammatory cells in the pleural biopsies.

		PLTB	NSP
Inflammatory cells	CD3	1549.0±261.9***	328.8±52.0
	CD4	1329.0±213.8*	768.9±144.5
	CD8	232.0±56.0	165.8±33.7
	CD8 / CD4	0.42±0.2	1.05±0.33
	CD68	398.1±59.2	595.9±225.0
	Neutrophil elastase	21.2±16.3	75.7±49.7
	Eosinophil granulocytes (hematoxylin and eosin staining)	100.0±27.7	65.2±19.3
	Mast cells (toluidine blue staining)	1.26±0.91**	51.96±29.14
	Mast cells (tryptase+)	0.8±0.5**	29.3±11.7
Th1 markers	IFN-γ	281.5±89.8	97.6±37.4
	CXCR3	2842.0±484.1	2218.0±422.5
	CCR5	1607.0±314.7	2092.0±427.4
	STAT4	329.0±75.7	359.9±127.0
	T-bet	33.2±17.9	10.8±5.7
	T-bet / GATA-3	0.099±0.08	0.015±0.009
Th2 markers	CCR4	5.41±2.27*	0.45±0.45
	CRTH2	2305.0±689.6	1900.0±364.9
	GATA-3	107.3±78.0***	560.8±152.4
	STAT6	13.9±13.9	1.4±1.4
Tregs marker	FOXP3	68.7±38.2	5.9±1.8

Data are presented as means (±SE).

*p<0.05, significantly different from NSP group,

**p<0.01, significantly different from NSP group,

***p<0.001, significantly different from NSP group.

### Immunohistochemical count for inflammatory cells

**Figure 3:**
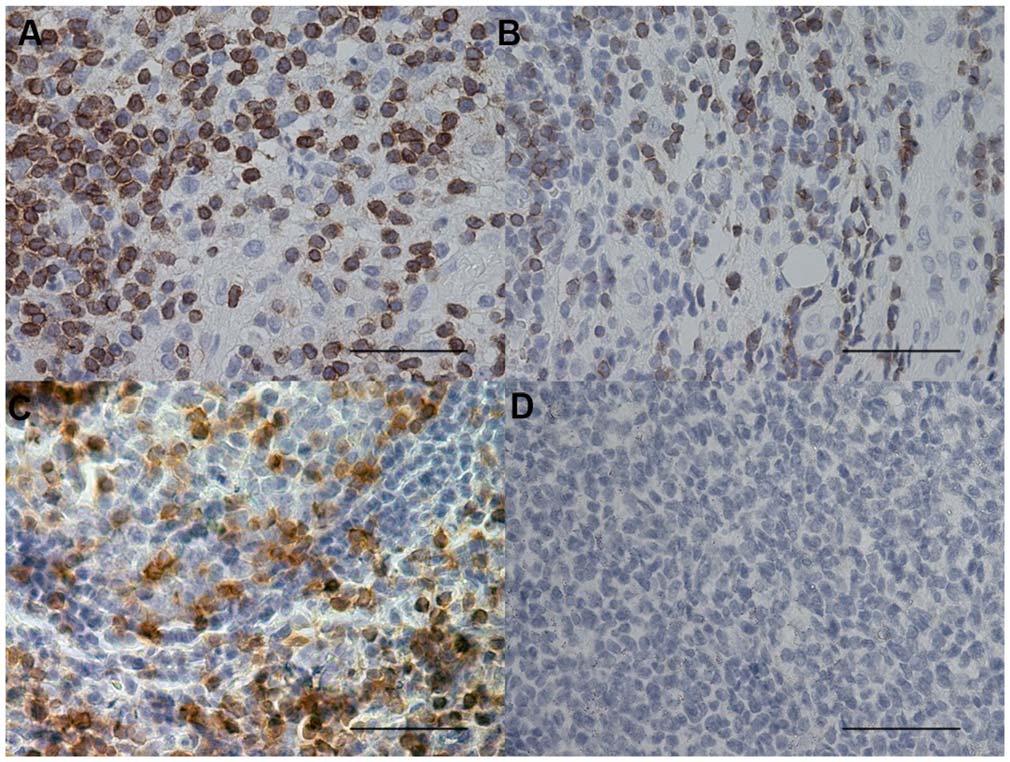
Photomicrographs showing the parietal pleura (A and B) and control tonsil (C and D) immunostained for identification of CD3+ cells (A–C). Photomicrographs D shows negative control slide of tonsils stained with normal nonspecific immunoglobulins of the same animal species of the primary antibody used at the same protein concentration as the primary antibody. CD3+ cells are stained in brown. Results are representative of those from 14 patients with PLTB (A) and 12 patients with NSP (B). Original magnification: 400×. The scale bar represents 50 µm.

**Figure 4:**
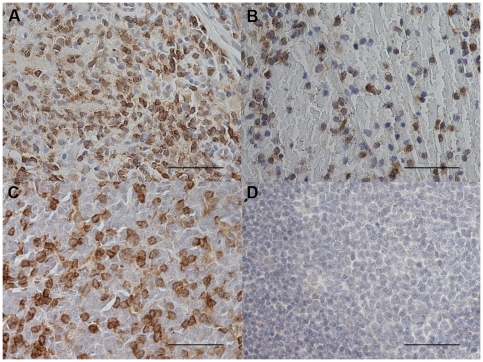
Photomicrographs showing the parietal pleura (A and B) and control tonsil (C and D) immunostained for identification of CD4+ cells (A–C). Photomicrographs D shows negative control slide of tonsils stained with normal nonspecific immunoglobulins of the same animal species of the primary antibody used at the same protein concentration as the primary antibody. CD4+ cells are stained in brown. Results are representative of those from 14 patients with PLTB (A) and 12 patients with NSP (B). Original magnification: 400×. The scale bar represents 50 µm.

**Figure 5:**
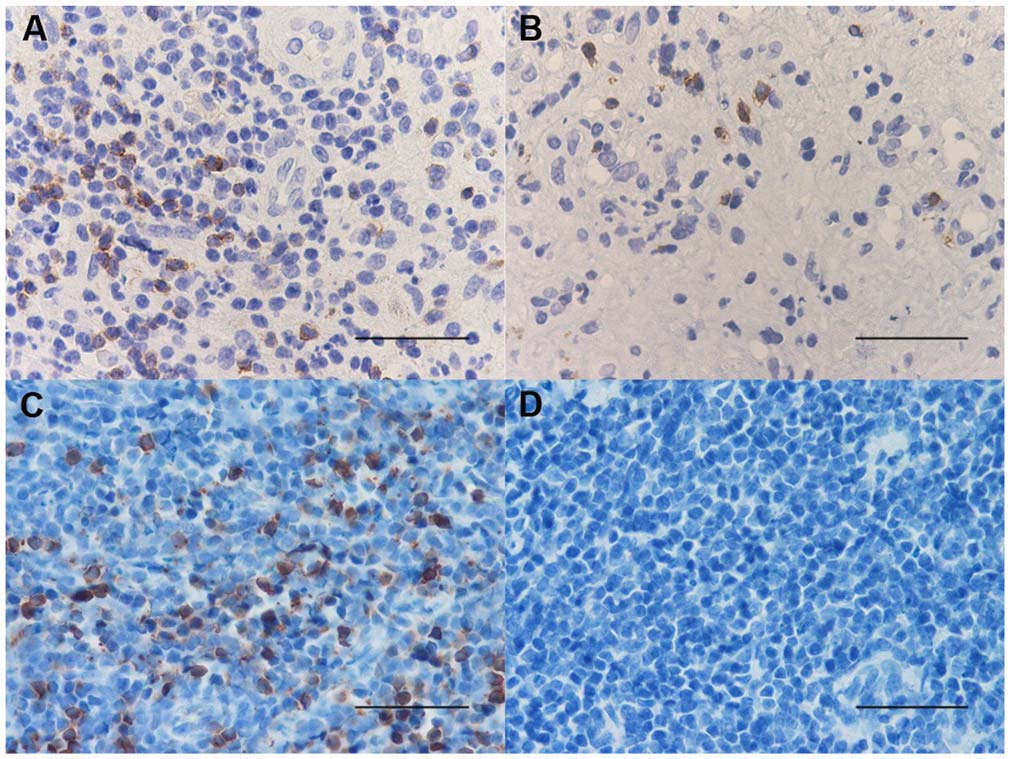
Photomicrographs showing the parietal pleura (A, B) and control tonsil (C and D) immunostained for identification of CD8+ cells (A–C). Photomicrograph D shows negative control slide of tonsils stained with normal nonspecific immunoglobulins of the same animal species of the primary antibody used at the same protein concentration as the primary antibody. CD8+ cells are stained in brown. Results are representative of those from 14 patients with PLTB (A) and 12 patients with NSP (B). Original magnification: 400×. The scale bar represents 50 µm.

**Figure 6:**
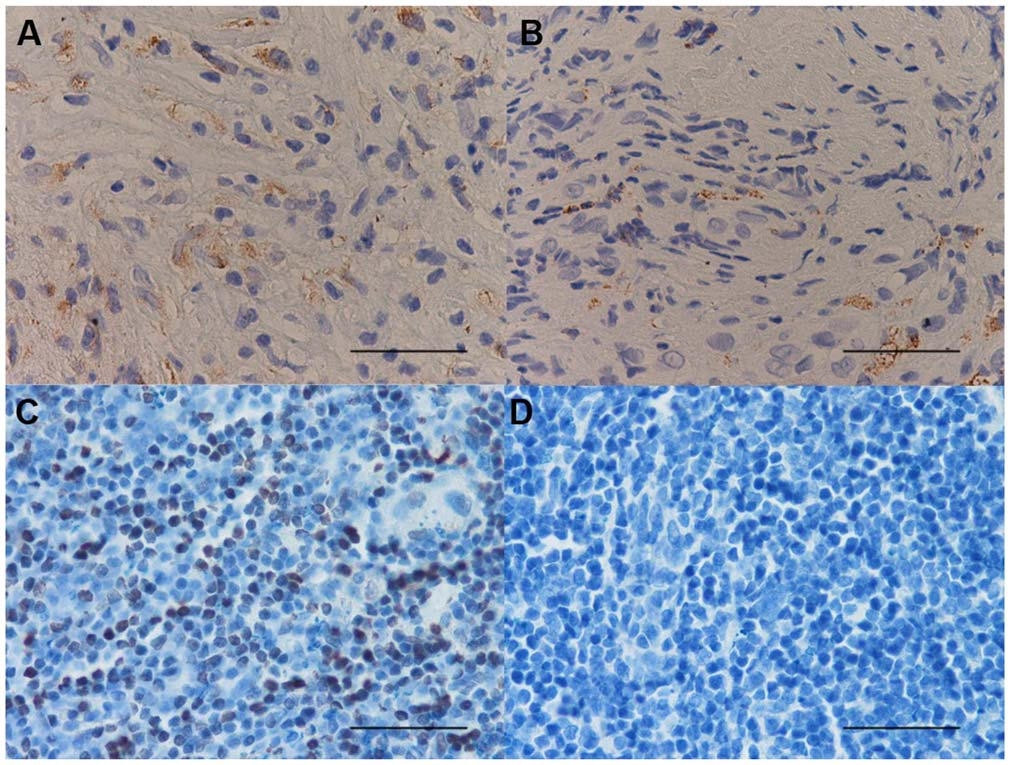
Photomicrographs showing the parietal pleura (A, B) and control tonsil (C and D) immunostained for identification of CD68+ cells (A–C). Photomicrograph D shows negative control slide of tonsils stained with normal nonspecific immunoglobulins of the same animal species of the primary antibody used at the same protein concentration as the primary antibody. CD68+ cells are stained in brown. Results are representative of those from 14 patients with PLTB (A) and 12 patients with NSP (B). Original magnification: 400×. The scale bar represents 50 µm.

**Figure 7:**
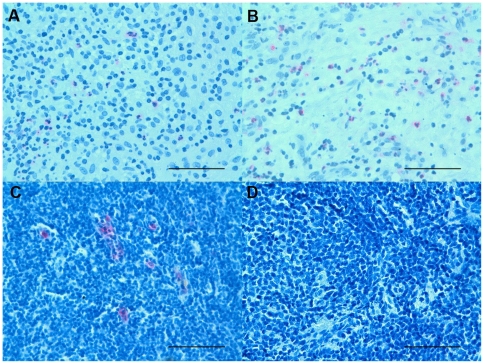
Photomicrographs showing the parietal pleura (A, B) and control tonsil (C and D) immunostained for identification of neutrophil elastase+ cells (A–C). Photomicrograph D shows negative control slide of tonsils stained with normal nonspecific immunoglobulins of the same animal species of the primary antibody used at the same protein concentration as the primary antibody. Neutrophil elastase+ cells are stained in red. Results are representative of those from 14 patients with PLTB (A) and 12 patients with NSP (B). Original magnification: 400×. The scale bar represents 50 µm.

The number of CD3+ and CD4+ cells was significantly increased in PLTB patients compared with NSP subjects (1549.0±261.9 vs 328.8±52.0 for CD3+ cells, p<0.001 and 1329.0±213.8 vs 768.9±144.5 for CD4+ cells, p = 0.04) (Table 1 and Figures 3 and 4). In contrast, the number of tryptase+ cells was significantly decreased in PLTB (0.8±0.5 vs 29.3±11.7, p = 0.008) (Figure 1 and Table 1). No significant differences were found in the number of CD8, CD68 and neutrophil elastase immunoreactive cells (Table 1 and Figures 5, 6 and 7).

### Immunohistochemical count for Th1, Th2 and Treg markers

**Figure 8:**
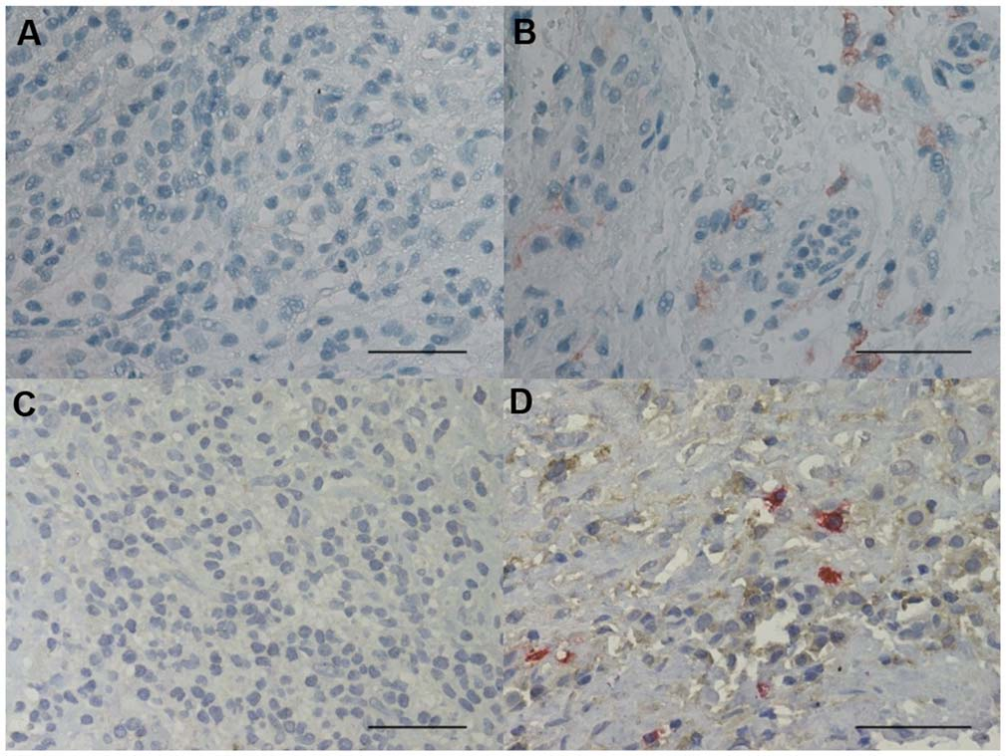
Photomicrographs showing the parietal pleura (A and B) immunostained for GATA-3+ cells or the parietal pleural (C and D) double immunostained for GATA-3+ cells and tryptase+ cells. GATA-3 is stained in red in A and B and brown in C and D. Tryptase is stained in red. Results are representative of those from 14 patients with PLTB (A and C) and 12 patients with NSP and (B and D). Original magnification: 400×. The scale bar represents 50 µm.

**Figure 9:**
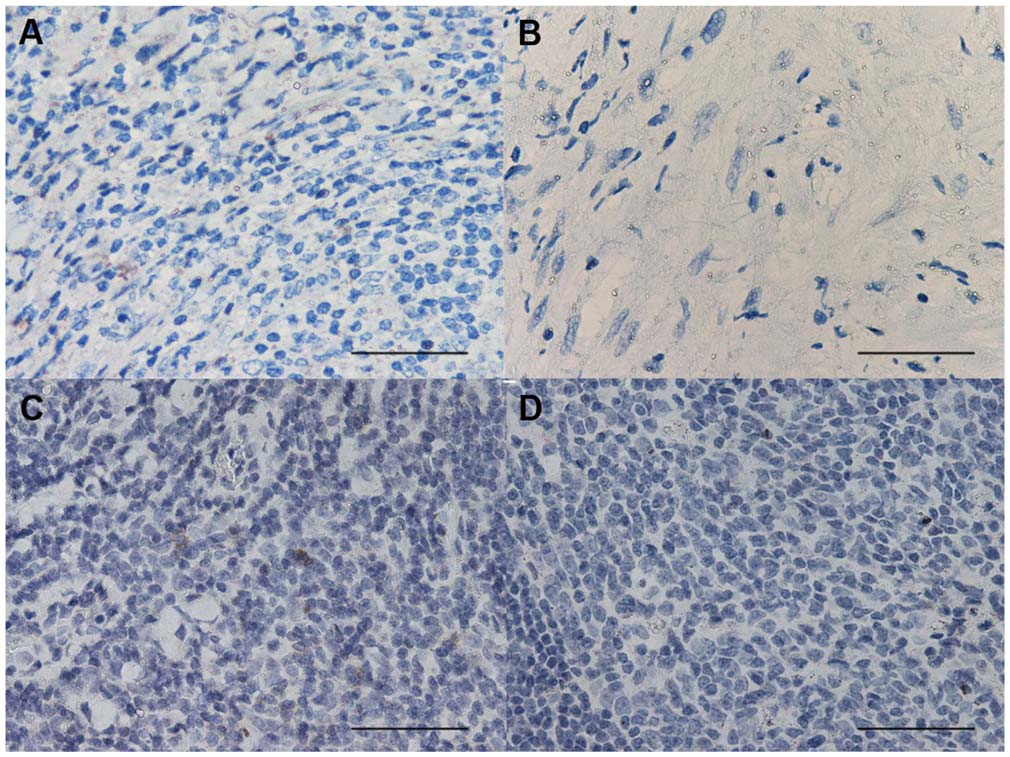
Photomicrographs showing the parietal pleura (A, B) and control tonsil (C and D) immunostained for identification of CCR4+ cells (A–C). Chemokine receptor CCR4+ cells are stained brown. Photomicrograph D shows negative control slide of tonsil immunostained using normal nonspecific immunoglobulins. Results are representative of those from 14 patients with PLTB (A) and 12 patients with NSP (B). Original magnification: 400×. The scale bar represents 50 µm.

**Figure 10:**
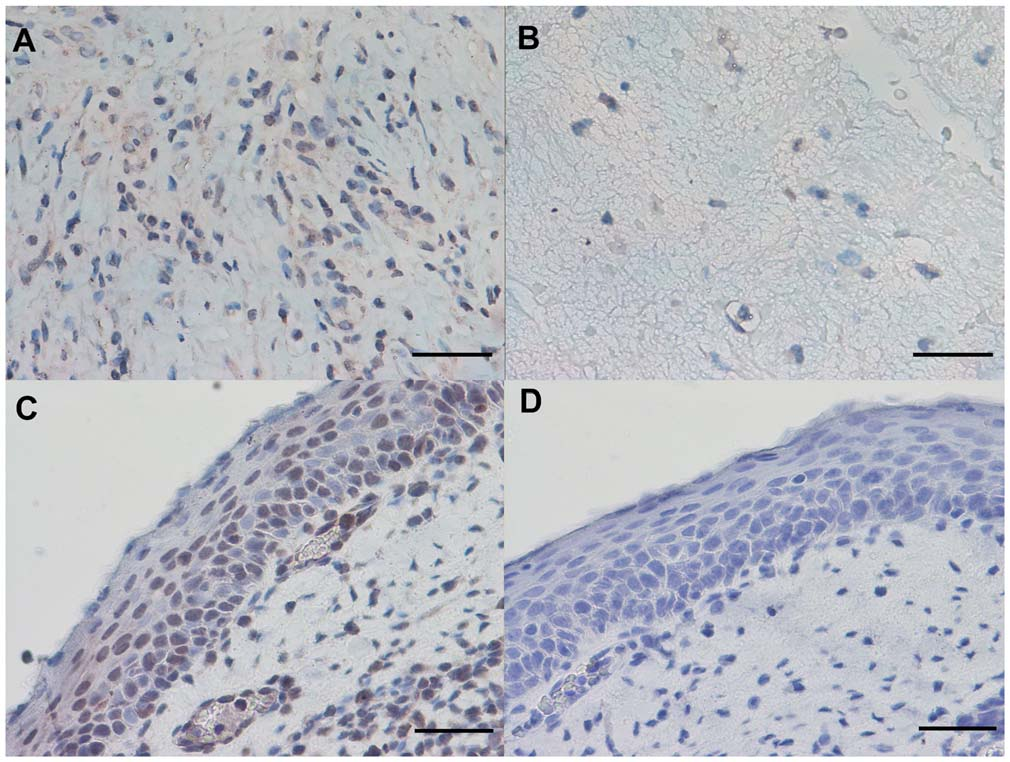
Photomicrographs showing the parietal pleura (A, B) and positive control oral mucosa (C and D) immunostained for identification of IFN-γ+ cells (A–C). IFN-γ+ cells are stained brown. Photomicrograph D shows negative control slide of oral mucosa surface epithelium immunostained using normal nonspecific immunoglobulins. Results are representative of those from 14 patients with PLTB (A) and 12 patients with NSP (B). Original magnification: 400×. The scale bar represents 50 µm.

**Figure 11:**
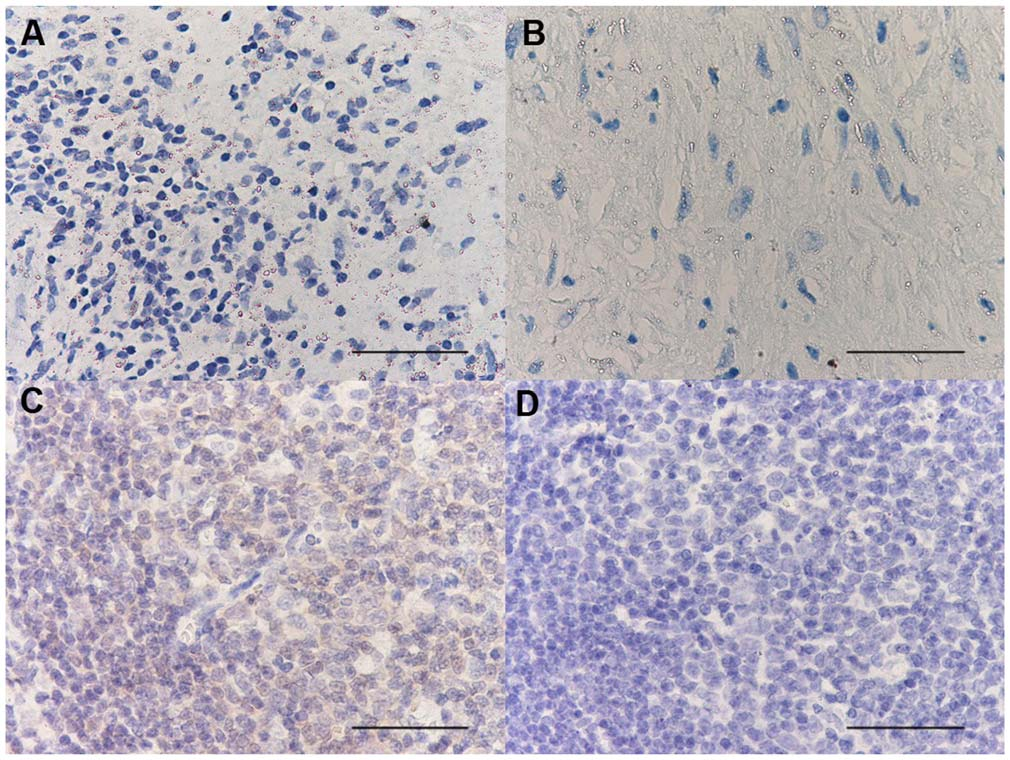
Photomicrographs showing the parietal pleura (A, B) and positive control tonsil (C and D) immunostained for identification of STAT6+ cells (A–C). STAT6+ cells are stained brown. Photomicrograph D shows negative control slide of tonsil immunostained using normal nonspecific immunoglobulins. Results are representative of those from 14 patients with PLTB (A) and 12 patients with NSP (B). Original magnification: 400×. The scale bar represents 50 µm.

**Figure 12:**
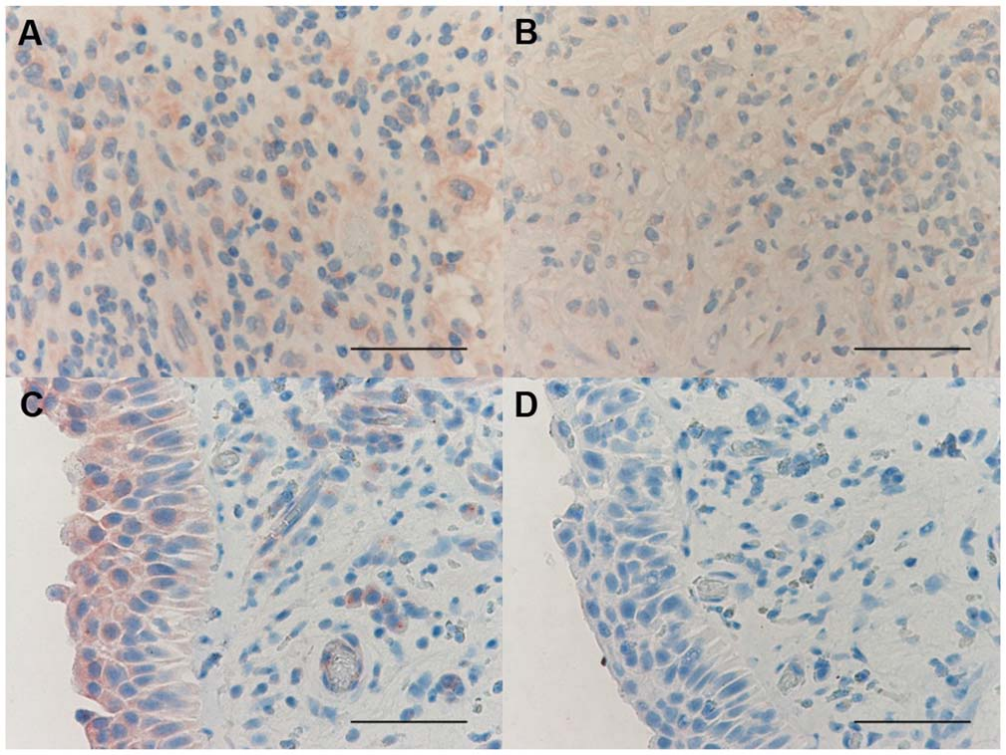
Photomicrographs showing the parietal pleura (A, B) and positive control nasal polyp (C) immunostained for CRTH2 (A–C). CRTH2+ cells are stained in red. Photomicrograph D shows negative control slide of nasal polyp immunostained using normal nonspecific immunoglobulins. Results are representative of those from 14 patients with PLTB (A) and 12 patients with NSP (B). Original magnification: 400×. The scale bar represents 50 µm.

**Figure 13:**
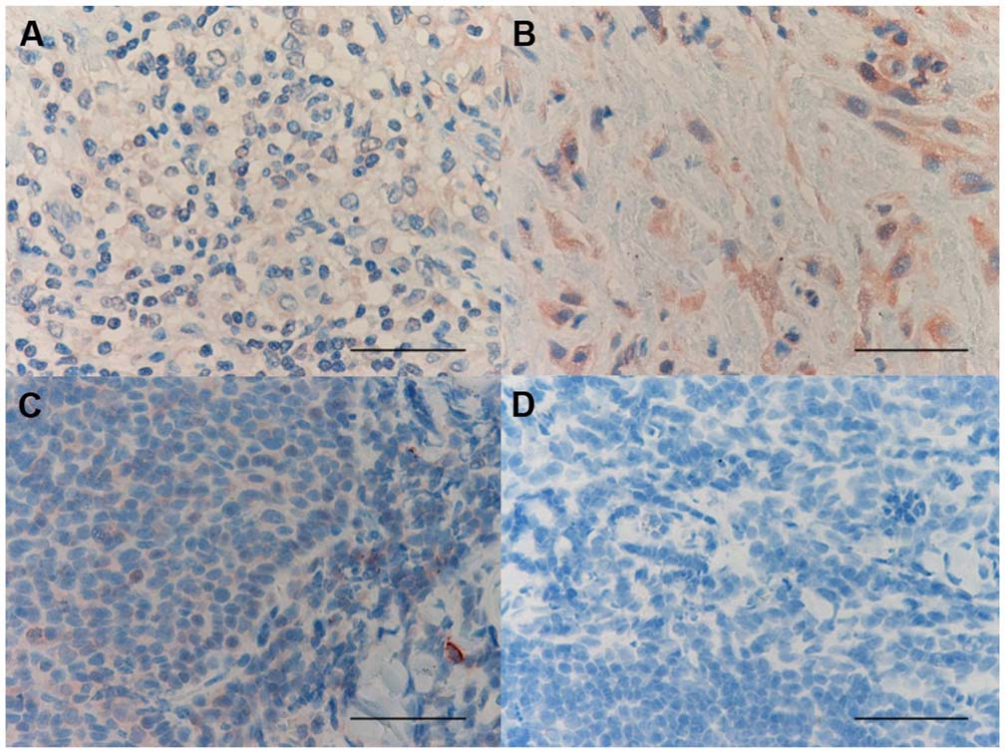
Photomicrographs showing the parietal pleura (A, B) and positive control tonsil (C) immunostained for CXCR3 (A–C). CXCR3+ cells are stained in red. Photomicrograph D shows negative control slide of tonsil immunostained using normal nonspecific immunoglobulins. Results are representative of those from 14 patients with PLTB (A) and 12 patients with NSP (B). Original magnification: 400×. The scale bar represents 50 µm.

**Figure 14:**
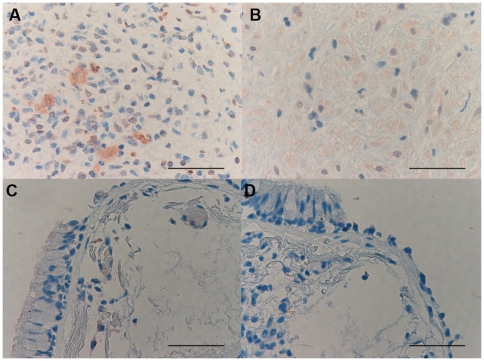
Photomicrographs showing the parietal pleura (A, B) and positive control bronchial biopsy (C) immunostained for CCR5 (A–C). CCR5 positive cells are stained in red. Photomicrograph D shows negative control slide of bronchial biopsy immunostained using normal nonspecific immunoglobulins. Results are representative of those from 14 patients with PLTB (A) and 12 patients with NSP (B). Original magnification: 400×. The scale bar represents 50 µm.

**Figure 15:**
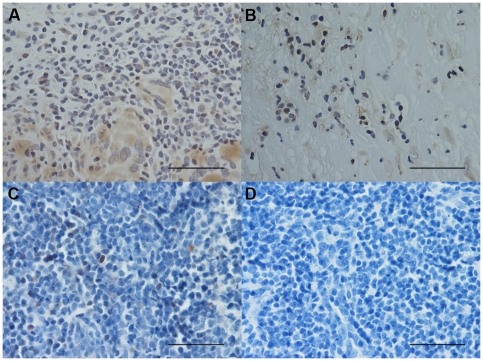
Photomicrographs showing the parietal pleura (A, B) and positive control tonsil (C) immunostained for identification of STAT4+ cells (A–C). STAT4, positive cells are stained brown. Photomicrograph D shows the negative control slide of tonsil immunostained using normal nonspecific immunoglobulins. Results are representative of those from 14 patients with PLTB (A) and 12 patients with NSP (B). Original magnification: 400×. The scale bar represents 50 µm.

**Figure 16:**
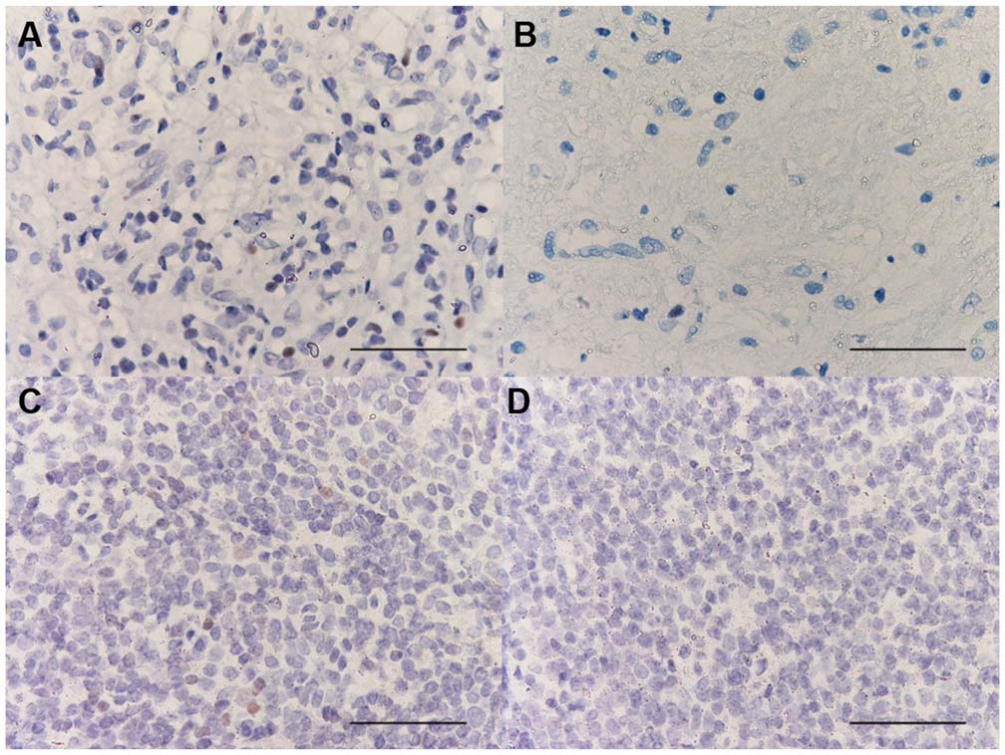
Photomicrographs showing the parietal pleura (A, B) and positive control tonsil (C) immunostained for identification of T-bet+ cells (A–C). T-bet positive cells are stained brown. Photomicrograph D shows the negative control slide of tonsil immunostained using normal nonspecific immunoglobulins. Results are representative of those from 14 patients with PLTB (A) and 12 patients with NSP (B). Original magnification: 400×. The scale bar represents 50 µm.

**Figure 17:**
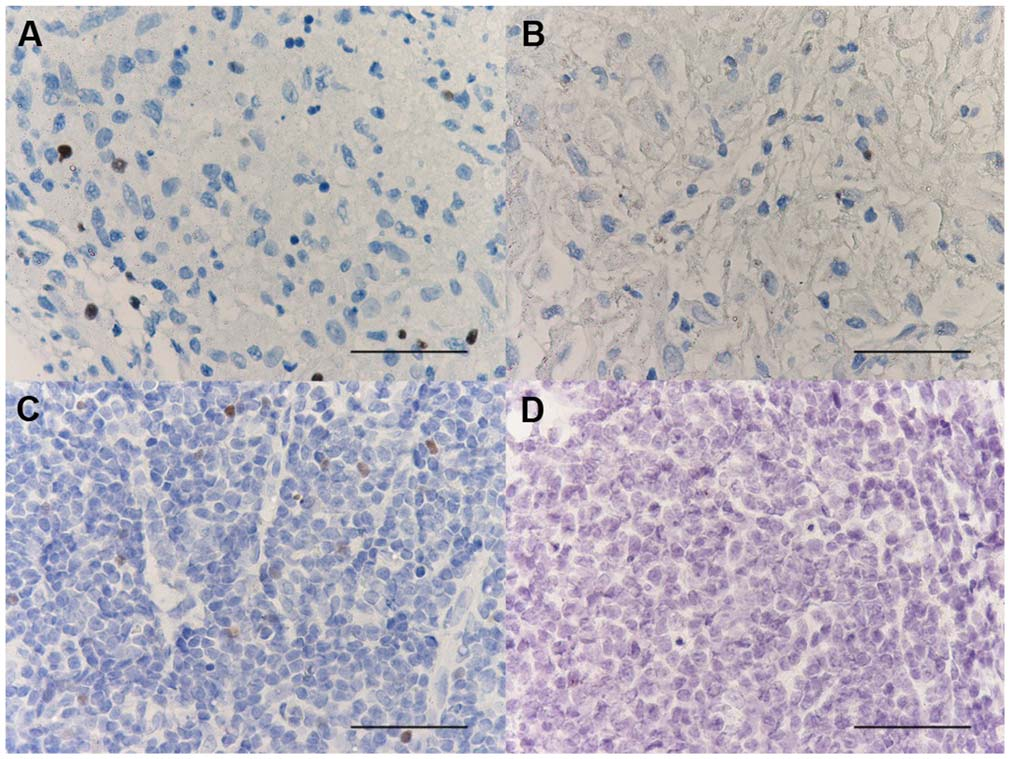
Photomicrographs showing the parietal pleura (A, B) and positive control tonsil (C) immunostained for identification of FOXP3+ cells (A–C). FOXP3 positive cells are stained brown. Photomicrograph D shows the negative control slide of tonsil immunostained using normal nonspecific immunoglobulins. Results are representative of those from 14 patients with PLTB (A) and 12 patients with NSP (B). Original magnification: 400×. The scale bar represents 50 µm.

The GATA-3+ cell number was significantly decreased in PLTB patients compared with NSP subjects (107.3±78.0 vs 560.8±152.4, p<0.001) (Table 1 and Figure 8). In addition, the CCR4+ cell number was significantly increased in PLTB patients (5.41±2.27 vs 0.45±0.45, p = 0.03) (Table 1 and Figure 9). There were no statistically significant differences in the number of IFN-γ, STAT6, CRTH2, CXCR3, CCR5, STAT4, T-bet, and FOXP3 immunoreactive cells between the two groups (Table 1 and Figures 10, 11, 12, 13, 14, 15, 16, and 17).

### CD4/CD8, Tbet/GATA3 and GATA-3/tryptase double-staining immunohistochemistry

**Figure 18:**
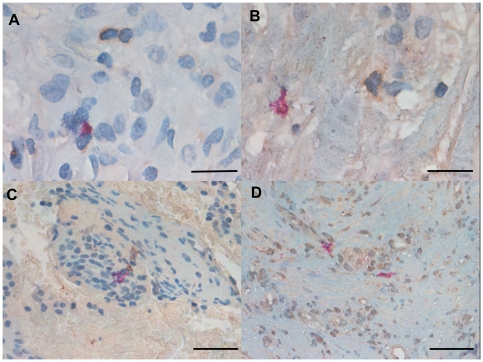
Photomicrographs showing the parietal pleura (A and B) double immunostained for CD4+ cells and CD8+ cells or the parietal pleural (C and D) double immunostained for Tbet+ cells and GATA-3+ cells. CD4 and Tbet are stained in red and CD8 and GATA-3 are stained in brown. Results are representative of those from 6 patients with PLTB (A and C) and 6 patients with NSP and (B and D). Original magnification: 1000× (A and B) and 400× (C and D). The scale bar represents 50 µm.

We were unable to detect co-localization of both tryptase and GATA-3 and Tbet and GATA-3 in the same cells in the parietal pleura in PLTB or NSP subjects (Figures 8 and 18). Around 10% of cells in the parietal pleura were both CD4 and CD8 positive without any significant differences between PLTB vs NSP subjects. The ratio of CD4/CD8 and Tbet/GATA-3 cells was not significantly different in the parietal pleura in PLTB compared with control NSP subjects (Table 1).

### Quantification of RORC2 and IFN-γ mRNA levels

RORC2 and IFN-γ mRNA levels were significantly increased in PLTB patients compared with NSP subjects (19.8±0.4 vs 18.0±0.3, p = 0.007; 16.8±0.2 vs 14.9±0.5, p<0.01 respectively).

### Overall accuracy of inflammatory cells and markers in the differential diagnosis

To analyze the diagnostic accuracy of clinical and laboratory parameters to predict PLTB, ROC analysis was performed for toluidine blue staining for mast cells detection, tryptase+ cells and GATA-3+ cells. Among the single markers, GATA-3 had the greatest AUC (0.88; 95% confidence interval [CI], 0.7197 to 1.037, p = 0.002). Compared to GATA-3 alone, the AUC for the combination of toluidine blue staining for mast cells, tryptase+ cells and GATA-3+ cells (AUC = 0.94; 95% CI 0.8475 to 1.028; p<0.001) was higher suggesting a better performance with the combination of the three markers in discriminating between PLTB and NSP. Despite the greatest AUC obtained for combination of the three markers compared to GATA-3 alone, the sensitivity and specificity showed a better performance for GATA-3 alone (cut off value of the combination 0.1995, sensitivity = 100% and specificity 75%; cut off value of GATA-3 alone 13.51, sensitivity = 75% and specificity 100%).

### Clinical and biological correlations

There was no significant correlation, between other clinical and biological data, including age or gender of the patients.

## Discussion

This is the first study providing a complete characterization of the inflammatory cell infiltrate in parietal pleural biopsies obtained from adult subjects from a non-endemic country, with a low prevalence of HIV infection, of untreated established PLTB compared with a control group of NSP.

PLTB patients have significantly increased numbers of CD3+ and CD4+ lymphocytes in their parietal pleura compared with NSP subjects in accordance with previous human and animal data [6]. However, a panel of Th1/Th2/Treg/Th17 markers was not particularly useful for the differential diagnosis of PLTB from NSP. This may reflect the fact that the T cell-driven inflammation in the parietal pleura of patients with PLTB is not sufficiently polarized towards a Th1 pattern and confirms previous data obtained on the pleural fluid [12]. Marchant and colleagues reported that most clones derived from tuberculous pleural effusions from untreated patients show a Th0 cytokine profile (production of both IFN-γ and IL-4), which only polarized towards a Th1 profile after 6 months of therapy and clinical healing [12]. In animal models of tuberculosis, the transcription factor STAT4, which is involved in Th1 development, is critical for mounting an effective Th1 immune response and clearance of *M.tuberculosis* from the lungs [17]. The absence of any differences in IFN-γ protein and STAT4 expression and activation in the parietal pleura in PLTB subjects further suggests the inability of patients to trigger either a sufficiently strong or early Th1-mediated immune response.

The presence of a small, but significant, increased number of CCR4+ cells in our PLTB patients is in keeping with previous data [13], [14] The up-regulation of CCR4 expression in the lungs in an animal model of tuberculosis [18] suggests the possibility of a Th2 response existing in PLTB. However, the CCR4 molecule is also expressed by Th subsets distinct from Th2 cells [19] and the expression of CCR4 on Th2 cells can be driven by GATA-3 in vitro [20]. These data suggest that CCR4 may be expressed on other T cell subsets in PLTB. For example most human Th17 clones express CCR4 in vitro [21]. Interestingly, we found an increased amount of RORC2, a marker of human Th17 cells, in the parietal pleura of PLTB patients compared to control group. Animal models suggest that whilst protection against *M.tuberculosis* infection does not require IL-17 or IL-23, IL-17 contributes to the maintenance of the inflammatory response and mycobacteria-specific Th17 cells may provide long-lasting immunity [22]. Future studies to better characterize the phenotype and the functional role of these CCR4+ and Th17 cells in the parietal pleura of patients with PLTB are warranted.

The absence of an increased number of CXCR3+ cells in the parietal pleura of subjects with PLTB is in keeping with previous data obtained in pleural effusions from patients with active PLTB [23]. The absence of a significant increase of the number of CCR5+ cells is more surprising and it is in contrast with previous studies performed in blood [22] and bronchoalveolar lavage (BAL) [24] of patients with active tuberculosis. However, in an animal model of tuberculosis, there is differential expression of these chemokine receptors in T-lymphocytes isolated from different lung compartments and most cells were both CXCR3+ and CCR5+ [25]. CCR5 is also expressed by both T-lymphocytes and monocytes/macrophages and in vitro CCR5 expression on these cells may be downregulated in the presence of high levels of its ligands, such as CCL3, CCL4 and CCL5, which are the major chemokines produced in response to *M.tuberculosis* infection in vitro [26].

Activation of FOXP3+ Treg cells may prevent an excessive inflammatory response to the host and/or may compromise the elimination of *M.tuberculosis*. Recent studies suggest an increased presence of Tregs in PLTB [10], [11] with the number of Treg cells in pleural fluid inversely correlating with local mycobacterial-specific immunity [27]. We speculate that the absence of significant differences in the FOXP3+ cell number between PLTB and NSP subjects, observed in our study, may be a marker of an excessive downregulation of the immune response against *M.tuberculosis* and that this may contribute to its delayed clearance.

We observed a number of toluidine blue+ cells significantly decreased in PLTB patients compared with NSP subjects. Toluidine blue stain for mast cells is able to distinguish patients with PLTB from the control group of subjects with NSP with a high sensitivity and specificity. Toluidine blue histochemical staining has a similar overall accuracy to tryptase immunostaining for mast cells and there was an almost complete overlap of the counts using these two different techniques. If confirmed, this test will be particularly useful in less affluent countries where there is the highest burden of PLTB and where *M.tuberculosis* culture and PCR are often unavailable due to their high cost.

In vitro studies and animal models show that activated mast cells participate to the immune response against *M.tuberculosis* and are abundant in human tuberculous lymphadenitis [28]–[30]. However, our data suggest that the subtype of pleural mast cells [31] may have a different functional role or are invaded and destroyed by *M.tuberculosis* [32].

There is also a decreased number of GATA-3+ cells in parietal pleura of PLTB patients. Although the main regulatory pathways for mast cell differentiation are not well characterized, there is increasing evidence for a role of GATA-3 in driving mast cell differentiation of uncommitted but differentiating lymphoid precursor cells [33]. Interestingly, *in-vitro* purified protein derivative (PPD)-stimulated peripheral blood mononuclear cells, obtained from active tuberculosis patients, have reduced GATA-3 mRNA levels [34]. Thus, we may speculate that *M.tuberculosis* induces GATA-3 downregulation in the parietal pleural cells during active PLTB [17]. We have been unable to detect tryptase and GATA-3 co-localization within the parietal pleural cells in either PLTB or NSP subjects. For this reason we speculate that the high number of GATA-3+ cells in the parietal pleura of the subjects with NSP may represent precursors of mast cells that are too immature to express tryptase and granules staining for toluidine blue. This hypothesis is in accord with the data from similar co-localization studies performed in the bronchial mucosa of asthmatic patients where the vast majority of GATA-3+ infiltrating immune cells were also CD3+ cells, with a very small fraction of double positive GATA-3+/tryptase+ cells [35]. Clearly this area requires further research both *in vitro* and *in vivo*.

In conclusion, untreated PLTB in patients from a non-endemic country with a low prevalence of HIV infection compared with a control group of subjects with NSP is characterized by the increased number of CD3, CD4, CCR4 and Th17 cells and by the presence of a decreased number of mast cells and GATA-3+ cells in the parietal pleura.

## Materials and Methods

### Ethics Statement

The study was approved by the ethics committee of the University-Hospital of Parma, Italy. It has been performed on archival paraffin-embedded specimens of parietal pleura collected by medical thoracoscopy between 1996–2006. These procedures were performed to diagnostic purposes in subjects who were admitted in the Respiratory Diseases and Thoracic Endoscopy Unit, Parma Hospital. At the end of diagnostic procedures, biopsies were archived in the Department of Pathology of Parma Hospital. The local Ethic-Committee has approved this study and informed consent has been judged not necessary because of the impossibility to contact all the patients that underwent the procedure in the past years. The ethics committee specifically waived the need for consent also because in this study the anonymity of the patient is preserved and no genetic studies are performed.

### Subjects

The clinical and microbiological characteristics of subjects obtained from our patient records are reported in Tables 2 and 3. The age was significantly higher in NSP patients compared with PLTB group (68.8±2.4 vs 45.1±4.3 years, p = 0.002). Diagnosis of PLTB and NSP was independently confirmed by three pathologists reviewing microbiological, histological, and molecular biology characteristics. All samples were obtained from subjects not treated with anti-tubercular therapy and with an undiagnosed exudative pleural effusion submitted for medical thoracoscopy at the Respiratory Diseases and Thoracic Endoscopy Unit, Parma Hospital, Italy.

**Table 2:** Clinical and demographic characteristics of the subjects.

	PLTB	NSP
Number of subjects	14	12
Age (years)	45.1±4.3	68.8±2.4*
Gender (M/F)	10/4	10/2
Pleural effusion side (right/left/bilateral)	7/7/0	5/5/2
PPD test (+/−)	8/1	1/2
Presence of fever at admission (yes/no)	12/2	5/7
Ethnic group	Caucasian 7African 2Asiatic 5	Caucasian 12

Data are presented as means (±SE).

*p<0.01 significantly different from NSP group.

**Table 3:** Microbiological characteristics of the subjects (detection of Mycobacterium tuberculosis ).

		Sputum			Pleural fluid			Bronchial washing			Pleural biopsy			
	Number of subjects	AFB+	Culture+	PCR+	AFB+	Culture+	PCR+	AFB+	Culture+	PCR+	AFB+	Culture+	PCR+	Caseous necrosis+
PLTB	14	1/6	0/3	0/2	0/10	0/10	5/11	0/3	0/4	0/5	0/11	4/8	7/8	12/14
NSP	12	ND	ND	ND	0/7	0/7	0/5	0/4	0/1	ND	0/1	ND	0/3	0/12

PLTB: pleural tuberculosis, NSP: nonspecific pleuritis, AFB: acid fast bacilli, PCR: polymerase chain reaction, ND: not determined.

Medical thoracoscopy was performed with patient spontaneously breathing under local anesthesia in the endoscopy suite according to a standardized method [36], [37].

Pleural fluid was analyzed for pH, biochemical markers, Gram and Ziehl-Nielsen stains, bacterial and mycobacterial cultures, as well as for the differential white blood cells count and cytopathological examination.

Pleural biopsies were used for Ziehl-Nielsen staining and *M.tuberculosis* conventional culture in addition to the radiometric mycobacterial culture system (BACTEC, Becton Dickinson, USA). PCR for detection of *M.tuberculosis* in formalin-fixed paraffin/embedded parietal pleura was performed as previously described [38].

Pleural biopsies were freshly fixed in 4% buffered formaldehyde and processed to paraffin wax. Serial sections 6 µm thick were cut firstly for histochemical analysis and subsequent 4 µm serial sections were cut for immunohistochemical analysis and were placed on charged slides as previously reported [39].

### Histochemical staining for mast cells and eosinophil granulocytes in pleura

In order to detect mast cells and eosinophil granulocytes, two serial sections from each subject included in the study were stained respectively with toluidine blue [2% toluidine blue in 0.7 M hydrochloric acid at pH 2.7; Sigma, St. Louis, MO [40]] and hematoxylin and eosin (H/E).

### Immunostaining in pleura for the other inflammatory cells, Th1, Th2 and Treg markers

Single immunohistochemical staining was performed on serial sections of parietal pleura biopsies [39]. The primary antibodies used, their unmasking conditions, the detection kits, the chromogen used and the positive controls are summarized in Tables 4 and 5. After deparaffinization and rehydration to expose the immunoreactive epitopes, endogenous peroxidase activity was blocked by incubating slides in 3% hydrogen peroxide in distilled water followed by washing in distilled water. Cell membranes were permeabilized with 0.1% saponin. Non-specific labeling was blocked by coating with blocking serum for 20 minutes at room temperature. The sections were incubated for 1 hour at room temperature with the primary antibody.

**Table 4:** Summary of the immunohistochemical procedures used to detect the other inflammatory cells.

Antibody specificity	Company Manufacturer	Catalogue code	Source/host	Dilution	Unmasking procedure	Secondary antibody and amplification step	Chromogen	Positive control
CD3	DAKO	A0452	Rabbit	1∶75, solution of 600 µg/mL	Microwave, 50 mM EDTA, pH 8	Vectastain Elite kit	DAB	Tonsil
CD4	Novocastra (now Leica Mycrosystems)	NCL-CD4-1F6	Mouse	1∶50 solution of 50 µg/mL	Microwave, 50 mM EDTA, pH 8	Ultravision LP kit	DAB	Tonsil
CD8	Thermo-scientific	RM-91160	Rabbit	1∶50 solution of unknown concentration	Microwave, W-CAP TEC Buffer pH 8 Bio-Optica	Vectastain Elite kit	DAB	Tonsil
CD68	DAKO	M0876	Mouse	1∶100 solution of 40 µg/mL	Microwave, 10 mM citrate, pH 6	Vectastain Elite kit	DAB	Tonsil
Neutrophil elastase	DAKO	M0752	Mouse	1∶50 solution of 110 µg/mL	Trypsin 0.125% solution	Vectastain Elite kit	Fast Red TR/Naphtol AS-MX	Tonsil
Mast cell tryptase	DAKO	M07052	Mouse	1∶50 solution of 85 µg/mL	Trypsin 0.125% solution	Ultravision LP	Fuchsin substrate chromogen system	Tonsil

W_CAP TEC = wax capture antigen retrieval solution code 15-6315/F from Bio-Optica (www.bio-optica.it); Ultravision LP Detection System is from Labvision (www.labvision.com) code Large Volume HRP Polymer (RTU) TL-125-HL; DAB: 3,3′-diaminobenzidine tetrahydrochloride.

**Table 5:** Summary of the immunohistochemical procedures used to detect the Th1, Th2 and Tregs cells.

	Antibody specificity	Manufacturer	Catalogue code	Source/ host	Dilution	Unmasking procedure	Secondary antibody and amplification step	Chromogen	Positive control
Th1 markers	IFN-γ	www.scbt.com	sc-8308	Rabbit	1∶150 solution of 200 µg/mL	Microwave, 10 mM citrate, pH 6	Vectastain Elite kit	DAB	Oral mucosa
	CCR5	Alexis Biochemicals (www.alexis-biochemicals.com)	ALX 210823	Goat	1∶300 solution of 2000 µg/mL	Microwave, 10 mM citrate, pH 6	Vectastain Elite kit	AEC	Bronchial biopsy
	CXCR3	R&D Systems (www.rndsystems.com)	MAB160	Mouse	1∶700 solution of 500 µg/mL	Microwave, 10 mM citrate, pH 6	Vectastain Elite kit	AEC	Tonsil
	STAT4	Atlas Antibodies (www.atlasantibodies.com)	HPA 001860	Rabbit	1∶50 solution of 70 µg/mL	Microwave, 50 mM EDTA, pH 8	Ultravision LP kit	DAB	Tonsil
	T-bet	www.scbt.com	sc-21749	Mouse	1∶800 solution of 200 µg/mL	Microwave, 10 mM citrate, pH 6	Vectastain Elite kit	DAB	Tonsil
Th2 markers	CCR4	Becton Dickinson (www.bd.com)	551121	Mouse	1∶100 solution of 500 µg/mL	Microwave, 10 mM citrate, pH 6	Vectastain Elite kit	DAB	Tonsil
	CRTH2	Cayman Chemicals (www.caymanchem.com)	10004886	Rabbit	1∶400 solution of 1000 µg/mL	Microwave, 10 mM citrate, pH 6	Vectastain Elite kit	AEC	Nasal polyp
	GATA3	www.scbt.com	sc-22206	Goat	1∶50 solution of 200 µg/mL	Microwave, 10 mM citrate, pH 6	Vectastain Elite kit	AEC	Nasal polyp
	STAT6	www.scbt.com	sc-621	Rabbit	1∶50 solution of 200 µg/mL	Microwave, 10 mM citrate, pH 6	ImmPRESS Anti-rabbit Ig (peroxidase) kit	DAB	Tonsil
Treg marker	FOXP3	eBioscience (www.ebioscience.com)	144-777	Mouse	1∶1600 solution of 500 µg/mL	Microwave, 50 mM EDTA, pH 8	ImmPRESS Anti-Mouse Ig (peroxidase) kit	DAB	Tonsil

Th1: T-helper type 1; Th2: T-helper type 2; Tregs: regulatory T cells; CCR: chemokine CC motif, receptor; CXC: chemokine CXC motif, receptor; T-bet: T box expressed in T cells; CRTH2: chemoattractant receptor-homologous molecule expressed on Th2; STAT: signal transducer and activator of transcription; GATA3: GATA motif binding protein 3; FOXP3: Forkhead box protein 3. AEC: 3-amino-9-ethylcarbazole; DAB: 3,3′-diaminobenzidine tetrahydrochloride.

For negative control slides, normal non-specific immunoglobulins (www.scbt.com) of the same animal species as the primary antibody were used at the same protein concentration. Positive control slides were included in each staining run for all the immunostaining performed. After repeated washing steps with PBS/0.1% saponin, the sections were subsequently incubated with secondary antibodies for 30 minutes at room temperature. After further washing steps, the sections were incubated with amplification reagents for 30 minutes at room temperature using a specific chromogen. Slides were counterstained in hematoxylin and mounted on aqueous or permanent medium.

### Double immunostaining in pleura for CD4/CD8, Tbet/GATA3 and GATA-3/tryptase

Six sections from PLTB patients and six sections from NSP subjects were respectively stained for CD4/CD8 or Tbet/GATA3 or GATA-3/tryptase prior to counterstaining with hematoxylin. The primary antibodies used, their unmasking conditions, the detection kits, the chromogen used and the positive controls are summarized in Table 6.

**Table 6:** Summary of the double staining immunohistochemical procedures.

Antibody specificity	Company Manufacturer	Catalogue code	Source/host	Dilution	Unmasking procedure	Secondary antibody and amplification step	Chromogen	Positive control
CD4	Novocastra (now Leica Mycrosystems)	NCL-CD4-1F6	Mouse	1∶50 solution of 50 µg/mL	Microwave, 10 mM citrate, pH 6	Vectastain Elite kit	Fast Red TR/Naphtol AS-MX	Tonsil
CD8	Thermo-scientific	RB-9009-P	Rabbit	1∶600 solution of 200 µg/mL	Microwave, 10 mM citrate, pH 6	Vectastain Elite kit	DAB	Tonsil
GATA-3	Santa Cruz	sc-22206	Goat	1∶50 solution of 200 µg/mL	Microwave, 10 mM citrate, pH 6	Vectastain Elite kit	DAB	Tonsil
T-bet	Santa Cruz	sc-21749	Mouse	1∶800 solution of 200 µg/mL	Microwave, 10 mM citrate, pH 6	Vectastain Elite kit	Fast Red TR/Naphtol AS-MX	Tonsil

DAB: 3,3′-diaminobenzidine tetrahydrochloride.

### Scoring system for histochemical and immunohistochemical staining

The presence of staining was determined by a single, blinded, investigator (LL) by microscopic examination. Stained cells were quantified in all sections. The image analysis was performed using an integrated microscope (Olympus, Albertslund, Denmark), video camera (JVC Digital color, JVC A/S, Tatstrup, Denmark), automated microscope stage (Olympus, Albertslund, Denmark) and PC running computer Image pro-Plus Software (Media Cybernetics; www.mediacy.com) to quantitate the area of staining. Results of the histochemical staining and of the single immunohistochemical staining are expressed as positive cells/mm^2^ and immunoreactive cells/mm^2^ respectively.

### Quantification of RORC2 and IFN-γ mRNA levels

Total RNA was extracted (RNeasy FFPE Kit, Qiagen) from two 10 µm thick sections of pleural biopsies and 1 µg used for cDNA synthesis. cDNA was synthesized using Omniscript RT kit (Qiagen) as per manufacturer's instructions. Primer pairs for human RORC2 (NR1F3, a Th17 marker) (Cat. n. QT01007685) and IFN-γ (Cat. n. QT01007685; a Th1 marker) were purchased from Qiagen. Quantitative real-time reverse transcriptase-polymerase chain reaction (RT-PCR) was carried out using Sybr-green (QuantiFast Sybr Green PCR kit cat. n. 204054, Qiagen) following manufacturer's protocol as previously described [41]. At the end of the RT-PCR run a melting curve analysis was carried out to verify that the cycle threshold (Ct) values were based upon a single PCR product (data not shown). Relative levels of cDNAs were established using the ΔΔCt methods against the housekeeping gene glyceraldehyde-3-phosphate dehydrogenase (GAPDH) (Cat. n.Hs_GAPDH_2_SG QuantiTect Primer Assay, Qiagen). After normalization, the value of ΔCt was subtracted from 45 (total number of RT-PCR cycles), thus higher ΔCt levels indicate higher mRNA levels.

### Statistical Analysis

Results are expressed as means ± SE. Comparisons between the two groups were performed with the nonparametric Mann–Whitney U test. Correlation coefficients were calculated with Spearman's rank method. In addition, receiver operator characteristic (ROC) analysis was performed and the area under the curve (AUC) was calculated separately for toluidine blue staining for detection of mast cells, tryptase+ cells and GATA-3+ cells to discriminate between NSP and PLTB subjects. Logistic regression (with ROC curve) was then used to identify the combination of the three markers. Overall accuracy was assessed with the use of a ROC curve, which plots the true positive rate (sensitivity) against the false positive rate (1-specificity). A P value of less than 0.05 was considered to indicate statistical significance. All reported P values are two-sided. Data analysis was performed using Prism 4 for Macintosh (v 4.0b, GraphPad Prism software inc., San Diego, California, USA).
